# Machine learning and multi-omics data in chronic lymphocytic leukemia: the future of precision medicine?

**DOI:** 10.3389/fgene.2023.1304661

**Published:** 2024-01-12

**Authors:** Maria Tsagiopoulou, Ivo G. Gut

**Affiliations:** ^1^ Centro Nacional de Analisis Genomico (CNAG), Barcelona, Spain; ^2^ Universitat de Barcelona (UB), Barcelona, Spain

**Keywords:** machine Learning, omics, multi-omics analysis, precision medicine, chronic lymphocytic leukemia (CLL), bioinformatics, NGS -next generation sequencing

## Abstract

Chronic lymphocytic leukemia is a complex and heterogeneous hematological malignancy. The advance of high-throughput multi-omics technologies has significantly influenced chronic lymphocytic leukemia research and paved the way for precision medicine approaches. In this review, we explore the role of machine learning in the analysis of multi-omics data in this hematological malignancy. We discuss recent literature on different machine learning models applied to single omic studies in chronic lymphocytic leukemia, with a special focus on the potential contributions to precision medicine. Finally, we highlight the recently published machine learning applications in multi-omics data in this area of research as well as their potential and limitations.

## 1 Introduction

The breakthroughs of Next-Generation Sequencing (NGS) over the last decade have led to an increase in both the volume and complexity of omics data in genome-wide (bulk) (Lander et al., 2001; Venter et al., 2001) and deeper at the single-cell level. NGS allowed the scientific community to study various biological mechanisms such as genetics (whole-genome sequencing), gene expression (RNA-seq), and epigenetics [DNA methylation (e.g., whole-genome bisulfite sequencing), chromatin accessibility (ATAC-seq), chromatin immunoprecipitation assays with sequencing (e.g., ChIP-seq for histone markers)] resulting in high dimensional omics data (Reuter et al., 2015). Apart from the genome-wide approaches, single-cell technologies provide the opportunity to study different modalities such as gene expression (scRNA-seq) and chromatin accessibility (scATAC-seq) at the resolution of individual cells (Heumos et al., 2023). This technology shows distinct advantages over bulk data, particularly in capturing the clonal architecture and the cell type composition of the tumor microenvironment.

In addition, global scientific communities and consortia such as The Cancer Genome Atlas (TCGA) (Tomczak et al., 2015), the International Cancer Genome Consortium (ICGC) (International Cancer Genome et al., 2010), BLUEPRINT (Martens and Stunnenberg, 2013), Human Cell Atlas (HCA) (Lindeboom et al., 2021), etc., make relevant results available to everyone by publishing omics data and metadata, giving the opportunity for further exploration and data integration. This vast amount of complex omics data can be analyzed with machine learning (ML) algorithms to uncover biomarkers or predictive signatures for better patient stratification and treatment selection. However, most of the published applications of ML are based on single omic studies such as only bulk gene expression. Although multi-omics analysis using ML is still at an early stage, many review articles have discussed how useful and significant the applications of ML in this area can be (Reel et al., 2021; Arjmand et al., 2022; Kharb and Joshi, 2023).

There are certain ML methods suitable for combining different modalities of omics data such as autoencoders which can reduce the multi-omics dimensionality and identify important patterns of the input data (Feldner-Busztin et al., 2023). A successful application from (Chaudhary et al., 2018) identified multi-omic features linked to the differential survival of patients with hepatocellular carcinoma by creating two subgroups. Applications like this are a transformative step forward in the domain of personalized oncology.

Here we focus on multi-omics data in the context of chronic lymphocytic leukemia (CLL), along with the application of ML techniques, and the potential of this emerging field. CLL is an ideal model for the integration of multi-omics data using ML methodologies since it is the most common adult leukemia in Western countries (Kipps et al., 2017) and is characterized by clinical and biological heterogeneity (Delgado et al., 2020). Interestingly, this heterogeneity is reflected in a complex interaction between genetics, epigenetics, and the tumor microenvironment (Delgado et al., 2020) making it a promising area of multi-omics applications. The vast majority of the publications applying ML in CLL are related to clinicobiological data or single omic studies data set. Since few publications exist with multi-omic data and ML in CLL (Argelaguet et al., 2018; Lu et al., 2021; Tsagiopoulou et al., 2022), we discuss them in addition to single omic studies showing the significance of this application, the promising field of ML and multi-omic data.

## 2 Precision medicine and single omic studies in CLL

### 2.1 Precision medicine in CLL

Before the applications of NGS and omics data, the patient’s stratification and clinical management of CLL were performed with technologies such as fluorescence *in situ* hybridization (FISH) and targeted sequencing. The first important component reported in CLL was the importance of cytogenetic abnormalities which, ranking from high to low risk, are del(17p), del(11q), trisomy 12 and del(13q) (Baliakas et al., 2016). In addition to del17p, resulting in *TP53* gene inactivation, the TP53 mutations are associated with a short time to progression and, consequently, an early need for treatment, poor response to chemoimmunotherapy and an overall dismal outcome (Dohner et al., 2000; Zenz et al., 2010). The classification of CLL cases based on somatic hypermutation (SHM) status of the clonotypic BCR IG became the strongest prognostic marker in CLL until now (Fais et al., 1998; Hamblin et al., 1999; Chiorazzi and Ferrarini, 2003). This distinction includes two categories with markedly different outcomes: those with little or no somatic hypermutation (SHM) (‘unmutated CLL’, U-CLL) who follow considerably more aggressive clinical courses compared to those with a significant SHM burden (‘mutated CLL’, M-CLL).

However, NGS empowers precision diagnostics in CLL, introducing other genomic markers with prognostic or predictive impact. Whole-genome sequencing studies revealed important new driver mutations in *MYD88*, *NOTCH1*, *SF3B1*, *POT1*, and *XPO1*, which were associated with clinical outcomes (Knisbacher et al., 2022). Moreover, sub-clonal *TP53* mutations with variant allele frequency (VAF) below 10% were impossible to detect before NGS. This undetected mutation contributed to relapse after chemoimmunotherapy. Nowadays, *TP53* mutations are considered for targeted therapies such as *BTK* inhibitors (Byrd et al., 2013) serving as a predictive marker for treatment outcomes.

Several targeted therapies with remarkable clinical efficacy are on the market but still resistance and relapse occur. The challenge for precision medicine is the definition of more predictive markers that will assist in the better stratification of the patients and clinical decision-making.

### 2.2 Single omic studies in CLL and applications of machine learning

**FIGURE 1:**
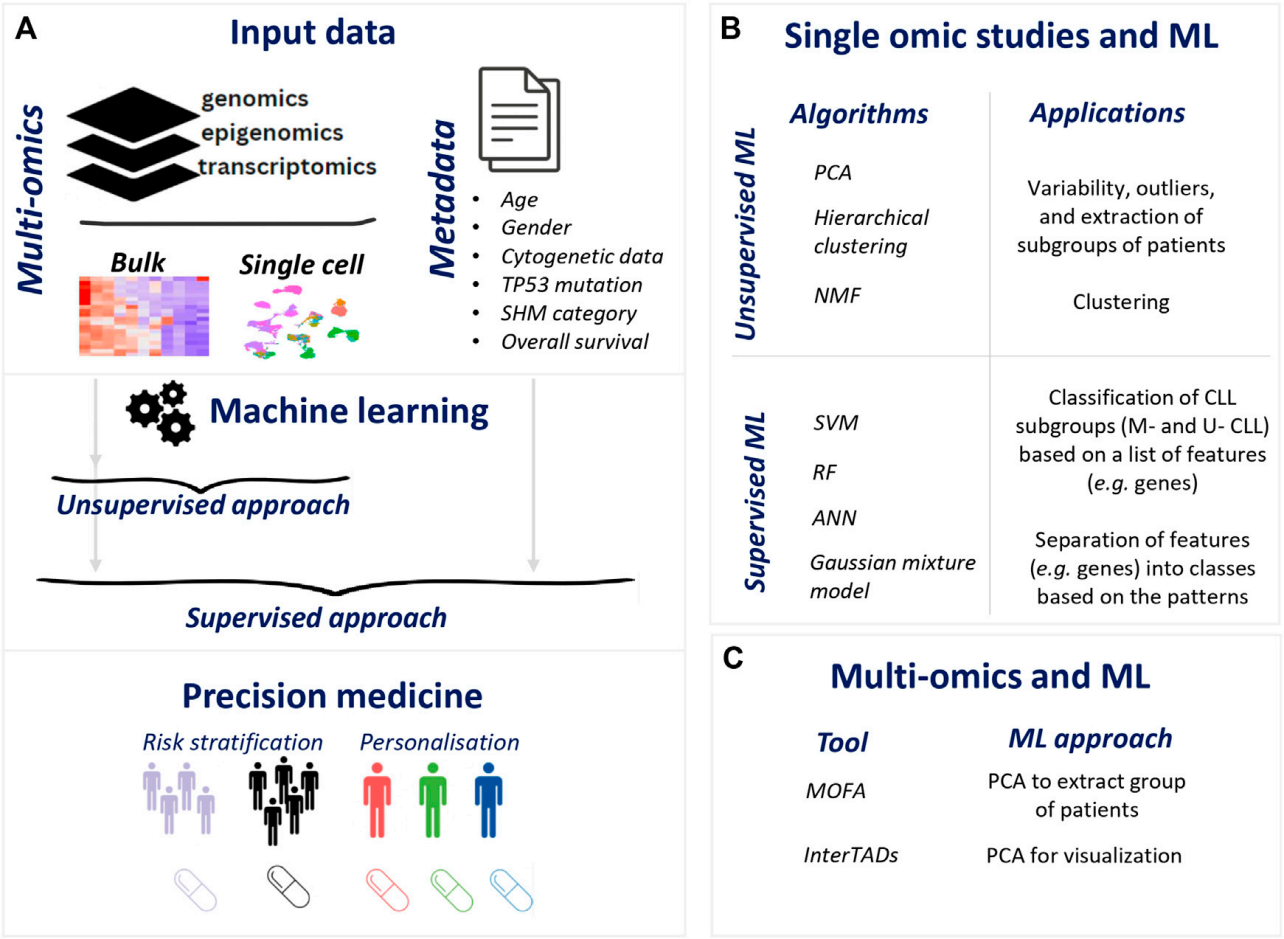
Illustration of ML for precision medicine in CLL. **(A)**. Flow of ML application in omics data resulting precision medicine applications **(B)**. ML application in single omic studies **(C)**. ML methods in multi-omics in CLL.

There are two primary ML strategies: supervised and unsupervised. The main difference between them is the requirement of metadata information to label the training data in a supervised manner, whereas unsupervised methods are based on the raw data only (Xu and Jackson, 2019) (Figures 1A,B).

The most popular ML techniques for analyzing single omic studies data in CLL are the principal component analysis (PCA) and hierarchical clustering which are unsupervised methods using metadata information for visualization purposes. PCA transforms high-dimensional data into a lower-dimensional space (Jolliffe and Cadima, 2016) and is used for data exploration such as variability, outliers, and extraction of subgroups of patients. Another popular methodology is hierarchical clustering which separates the variables [patients or events (genes, chromosomal locations)] into clusters based on their distances (Johnson, 1967). Plenty of single omic studies use the data to evaluate the separation of the subgroups of interest. For example, CLL patients with mutated *IGHV* genes (M-CLL) and patients with unmutated *IGHV* genes (U-CLL) were found to be divided using PCA and hierarchical clustering in a range of omics data such as gene expression, DNA methylation, histone markers, and chromatin accessibility (Kulis et al., 2012; Beekman et al., 2018). Diving into U-CLL subgroup, cases carrying identical BCR belonging to an aggressive subgroup of the disease called stereotyped subset #8 were found to be distinct using PCA and hierarchical clustering in different single omic modalities, *i.e.,* gene expression, DNA methylation, and histone modifications (Papakonstantinou et al., 2019; Tsagiopoulou et al., 2023). Trisomy 12 showed a distinct DNA methylation pattern together with altered chromatin activation using PCA explains some of the biological differences of this cytogenetically defined subtype (Beekman et al., 2018; Tsagiopoulou et al., 2020). An additional layer of analysis is the *k*-means algorithm (MacQueen, 1967) that has been applied in the CLL omics area in terms of the segregation of patients or features (genes, chromosomal location, etc.) (Chuang et al., 2012; Beekman et al., 2018) into *k* clusters.

Non-negative Matrix Factorization (NMF) is mainly used to perform unsupervised analysis of the complete set of genomic data (Kasar et al., 2015; Robbe et al., 2022). This method divides the data into two or more non-negative matrices, capturing meaningful mutational patterns in the data and uncovering distinct subgroups (Lee and Seung, 1999). One of these applications (Robbe et al., 2022) highlighted the key role of the noncoding mutation in the NMF which dramatically increases the level of distinction. Another publication using Bayesian NMF (weighted average AUC = 0.88) and 603 RNA-seq samples reported eight expression subgroups of CLL patients that were associated with the three epigenetic subtypes (n-, m-, i- CLL) and SHM status (Knisbacher et al., 2022).

Beyond unsupervised applications, supervised machine learning algorithms such as support vector machines (SVM) are commonly used on omics data, as it separates the different variables into different classes based on selected features (Cortes and Vapnik, 1995). These ML methodologies aim at providing novel perspectives on the classification and stratification of CLL cases. A table featuring all these ML applications in CLL is presented in Table 1. In more detail, a breakthrough application of SVM from (Queiros et al., 2015), used 133 DNA methylation CLL samples to build a prediction model (error rate = 1%) based on 5 CpG sites that classify CLL patients into three subgroups, namely, naive B-cell-like (n-CLLs), intermediate (i-CLLs), and memory B-cell-like CLL (m-CLLs), showing significant differences in the outcome. Since this classifier was built using Illumina 450K arrays and one of the five CpG sites in the classifier is not present in the EPIC array, a recent study published a new SVM classifier for these three epigenetic entities (Duran-Ferrer et al., 2020). Within the same study, a classifier was built using DNA methylation data (training series = 1,345 samples and external validation series = 711) along with SVM to diagnose an unknown B-cell tumor to the correct disease (e.g., CLL, MCL) and its subtypes (m-, n-, i- CLLs for CLL) (Duran-Ferrer et al., 2020). The classifier achieved remarkable accuracies for both the predictions of the main B-cell tumor entities (mean sensitivity was 97% for training series and 99% for validation series) and B-cell tumor subtypes (mean sensitivity was 90% for training series and 97% for validation series). In the same direction of diagnostic purposes, Artificial Neural Network (ANN) (Rumelhart et al., 1986), SVM, and Random Forest (RF) (Breiman, 2001) were evaluated for their performance in classifying healthy and CLL patients based on expression values of 12 genes (Shaabanpour Aghamaleki et al., 2019). These algorithms operate by analyzing the expression values of these genes and utilizing patterns within this data to classify the samples. Notably, ANN exhibited the highest accuracy with respect to the classification of CLL *versus* healthy samples (ANN = 0.969, SVM = 0.952, RF = 0.936). Between the two CLL subtypes the accuracy of ANN was 0.981.

**TABLE 1:** Classification machine learning models published in CLL studies.

Study	Omic measurement	ML method	Sample size	Performance Metrics
Knisbacher et al. (2022)	RNA-seq	Bayesian NMF	603 CLL cases	weighted average of AUC = 0.88
Queiros et al. (2015)	450K methylation arrays	SVM	133 CLL cases	classified 132/133 of the patients into the right epigenetic subgroup
				error rate (1.00%)
Duran-Ferrer et al. (2020)	450K and EPIC methylation arrays	SVM	Training series (*n* = 1,345): 809 cases of acute lymphoblastic leukaemia, 74 cases of mantle cell lymphoma, 490 CLL cases, 55 Diffuse large B cell lymphoma.	main B-cell tumor entities: mean sensitivity was 97% for training series and 99% for validation series
			External validation series (*n* = 711)	B-cell tumor subtypes: mean sensitivity was 90% for training series and 97% for validation series
Shaabanpour Aghamaleki et al. (2019)	gene expression microarrays	SVM, RF, ANN	42 CLL cases and 11 healthy controls	CLL and healthy donorts: SVM: AUC = 0.985, accuracy = 0.952/ RF: AUC = 0.969, accuracy = 0.936/ ANN: AUC = 0.991, accuracy = 0.969
				Two CLL subtypes: ANN: AUC = 0.991, accuracy = 0.981
Mosquera Orgueira et al. (2019)	RNA-seq	Gaussian mixture model and BigML	Training series (*n* = 196): 169 CLL, 22 monoclonal B cell lymphocytosis (MBL), and 5 small lymphocytic lymphoma (SLL) samples External validation series (*n* = 79): 72 CLL, 4 SLL, and 3 MBL samples.	90% precision at identifying patients that needed treatment in 5 years with 69.23% recall,
				88.57% precision at identifying patients without treatment in 5 years with 96.88% recall
				False positive rate = 3.1% False negative rate=30%
Rendeiro et al. (2020)	scRNA-seq	SVM	sample collection (day 0, 30, or 120/150) for each of the ∼19,000 single-cell transcriptomes for CLL cells from four donors.	cross-validated test set ROC-AUC values = 0.975 to 0.999

Regarding treatment prediction, a Gaussian mixture model (McLachlan, 2000) using expression data (initial cohort = 196 cases and validation cohort = 79 cases) from 2,198 genes. This model separates the different variables (e.g., genes) into different classes (Gaussian distributions) based on the patterns it finds in the data showing an association with the time to treatment. The genes associated with time to treatment were used for a ML classifier from BigML (BigML, 2011) and showed high accuracy in predicting the need for treatment within the first 5 years following diagnosis. (Mosquera Orgueira et al., 2019). This application paves the way for the identification of high-risk patients using ML.

Nowadays, state-of-the-art technologies to study individual cells (e.g., scRNA-seq) have required dimensionality reduction in their tools through unsupervised methods such as PCA or t-SNE. These techniques are used to project the single cells into lower dimensions. Even though the amount of scRNA-seq data in CLL has vastly increased in recent years, there is only one application of ML in downstream analysis using SVM (Rendeiro et al., 2020). This single-cell application of ML predicted the time point of sample collection (day 0, 30, or 120/150) after the start of ibrutinib therapy for 4 CLL patients using scRNA-seq data (cross-validated test set ROC-AUC values = 0.975–0.999). This suggests that single cells undergo changes that reflect the duration of ibrutinib therapy.

In summary, machine learning and single omic studies have significantly contributed to CLL research. Unsupervised methodologies such as PCA and hierarchical clustering have played a critical role in visualizing the landscape of CLL and finding patterns (Chuang et al., 2012; Kulis et al., 2012; Kasar et al., 2015; Beekman et al., 2018; Papakonstantinou et al., 2019; Tsagiopoulou et al., 2020; Robbe et al., 2022; Tsagiopoulou et al., 2023). These approaches help researchers explore and understand the complexities of the disease, enabling the classification of patients into distinct subgroups. ML classifiers offer a potential tool for disease diagnosis and disease subtype detection (Shaabanpour Aghamaleki et al., 2019; Duran-Ferrer et al., 2020), as well as treatment outcome prediction (McLachlan, 2000) which will help to identify high-risk patients and optimize treatment decisions. However, ML classifiers have not only distinguished different CLL subgroups but revealed new epigenetic-mediated categories including the high clinical relevance of the i-CLL subgroup (Queiros et al., 2015). In more detail, n-CLL and m-CLL cases were associated with IGHV mutational status and next omics studies associated the less well defined i-CLL group with a clinically aggressive subgroup of CLL called the stereotyped subset #2 (Bhoi et al., 2016) and a point mutation in IGLV3-21R110 (Nadeu et al., 2021). This classification of patients has been commonly used in research since then (Bhoi et al., 2016; Mallm et al., 2019) highlighting the importance of this model in precision medicine in CLL.

### 2.3 Applications of ML in multi-omic analysis in CLL

In terms of multi-omics data and ML, few publications took advantage of the rich data availability in CLL and by applying ML methodologies they offered new perspectives in CLL(Figure 1C).

A recently published tool for patient subtyping called Multi-Omics Factor Analysis (MOFA) used CLL as an application highlighting expected observations such as the importance of IGVH mutational status and reported new insights (Argelaguet et al., 2018). MOFA uncovers the principal components of biological and technical diversity when analyzing multiple omics datasets from the same samples. This method is based on PCA and uses the principal components to generate subgroups called factors. These factors can be shared by multiple omics modalities or can be datatype (single omic data) specific and enable a variety of downstream analyses, including the identification of subgroups and data imputation. The application of MOFA in CLL was based on DNA methylation and gene expression data from 200 patients. The results showed separation by known clinical markers and other unknown axes of variation such as oxidative stress. This observation was examined in more detail in the next publication of the same group, in which they discovered 6 factors/subgroups of CLL patients (Lu et al., 2021). Five out of six were associated with known markers (*i.e.,* IGVH mutational status, trisomy 12, the three epigenetic subtypes). However, they introduce a new Factor 4, previously unknown, as the “CLL proliferative drive” (CLL-PD). CLL-PD was associated with poor clinical outcome and with activation of mTOR-MYC-oxidative phosphorylation by gene expression, proteomic and single-cell resolution analysis. Except for the identification of the CLL subgroup based on ML, another methodology called InterTADs focuses on the integration of the multi-omic data considering the chromatin configuration of the genome (Tsagiopoulou et al., 2022). This method can detect topologically associated domains with different activity which is measured by mixing the values of the different omic data. Applying this approach across 135 CLL cases with paired gene expression and DNA methylation, meaningful results were reported in IGVH mutational status and trisomy 12. PCA and the metadata information of IGHV mutational status of the CLL patients were used to confirm the value of this approach since the explained variance of the PCs was increased compared to PCAs including the single omic datasets. These applications highlight the power of multi-omics data and ML to gain more granularity in the data and report new results with potential impact in precision medicine.

ML methodologies applied to multi-omics data offer fresh perspectives in the interpretation of diverse omics layers. These concepts often revolve around the identification of novel patient subgroups with distinct and uncharacterized characteristics, as exemplified by the MOFA tool (Argelaguet et al., 2018). Conversely, InterTADs (Tsagiopoulou et al., 2022) places a primary focus on genome regulation and activation, utilizing clinicobiological metadata for subgroup evaluation. It is worth noting that both of these tools have concentrated on a limited number of multi-omic datasets, typically three or fewer. In the future, the application of these or new methodologies should encompass a more comprehensive range of layers, covering both coding and noncoding regions.

### 2.4 Limitations in multi-omics and ML

Considering all the supporting evidence of single omic studies and multi-omics approaches that we discussed in the previous sections, the field of integration of multi-omics data using ML is a promising area supporting precision medicine. However, one significant limitation for the ML applications is the nature of omics data characterized by the high dimensionality (features) and small sample size (patients) (Hastie et al., 2009). The validation and reproducibility of ML applications with multi-omic data can be challenging, leading to the potential overestimation of model performance. This challenge becomes particularly pronounced when studying rare entities, such as stereotype subset #8 or cases carrying specific low-frequency genetic mutations, as it is exceptionally difficult to locate additional cohorts meeting the experimental conditions for validating ML findings.

ML heavily relies on data quality, and omics data can be noisy and subject to batch effects, potentially leading to biased or inaccurate results. In the same direction, multi-omics datasets are not comprehensive, as they often combine data from different sources. For instance, a dataset might include RNAseq data from blood samples and another omics layer from tissue biopsies which can present challenges when attempting to gather omics data exclusively from a single sampling site. This issue is less critical for malignancies originating in the blood, but even in such cases, cell sorting before analysis, while reducing background noise, can introduce bias due to factors such as cells undergoing apoptosis or releasing associated compounds. Finally, ML models may oversimplify the biological complexity of the studied systems, potentially missing relevant interactions and features especially in deep learning methodologies that are considered black-box models.

### 2.5 Future challenges and directions in multi-omics and ML

A future challenge involves the development of ML algorithms able to manage a large number of features within a small cohort. The development of the next-generation of ML multi-omics methodologies should consider the phenotypic outcome of gene expression as the primary link connecting various modalities of omics data. Moreover, there is no widely accepted approach for multi-omics data integration and a recent benchmarking paper (Cai et al., 2022) concluded that most of the tools did not show significantly enhanced performance over PCA, showing the importance of new approaches. The challenges associated with dimensionality, small sample sizes, and the high number of genes in omics data can be addressed with the introduction of single-cell data. Single-cell omics data, which focuses on individual cells rather than samples, offers a promising approach to overcome these limitations. This approach not only sidesteps the constraints of sample size but also provides valuable insights into gene regulation and activation, even if it does not directly address patient subtyping. Many applications of NMF in scRNA-seq in cancer reported diverse expression programs within the malignant cells including key features such as cell cycle and hypoxia showing a promising methodology for multi-omics studies (Barkley et al., 2022; Gavish et al., 2023). These results highlight the potential of ML methodologies in advancing single-cell multi-omics research.

## 3 Concluding remarks

The applications of ML and multi-omic data in CLL are still in their early stages, but the results highlighted by this review suggest that these technologies have the potential to significantly improve risk stratification and patient outcomes. However, this ongoing movement to multi-omics data utilizing ML methods will hopefully assist in truly implementing precision medicine for CLL patients in the near future.
